# A heteroscedastic hidden Markov mixture model for responses and categorized response times

**DOI:** 10.3758/s13428-019-01229-x

**Published:** 2019-03-28

**Authors:** Dylan Molenaar, Sandor Rózsa, Maria Bolsinova

**Affiliations:** 1 Department of Psychology, University of Amsterdam, Amsterdam, The Netherlands; 2 Washington University School of Medicine, St. Louis, MO USA; 3 ACTNext, Iowa City, IA USA

**Keywords:** Mixture models, Item response theory, Response times, Hidden Markov models

## Abstract

Various mixture modeling approaches have been proposed to identify within-subjects differences in the psychological processes underlying responses to psychometric tests. Although valuable, the existing mixture models are associated with at least one of the following three challenges: (1) A parametric distribution is assumed for the response times that—if violated—may bias the results; (2) the response processes are assumed to result in equal variances (homoscedasticity) in the response times, whereas some processes may produce more variability than others (heteroscedasticity); and (3) the different response processes are modeled as independent latent variables, whereas they may be related. Although each of these challenges has been addressed separately, in practice they may occur simultaneously. Therefore, we propose a heteroscedastic hidden Markov mixture model for responses and categorized response times that addresses all the challenges above in a single model. In a simulation study, we demonstrated that the model is associated with acceptable parameter recovery and acceptable resolution to distinguish between various special cases. In addition, the model was applied to the responses and response times of the WAIS-IV block design subtest, to demonstrate its use in practice.

In psychological and educational measurement of constructs and abilities, within-subjects differences may exist in the psychological processes that resulted in the responses to the items of the test. For instance, respondents may resort to fast guessing on some of the items of an educational measurement test but use a regular response process on the other items (Schnipke & Scrams, 1997); respondents may alternate between memory retrieval and actual calculation on the items of an arithmetic test (Grabner et al., 2009); or they may use trial and error on some items of a spatial puzzle but use an analytical strategy on others (Goldstein & Scheerer, 1941).

The objective of this article is to improve on existing statistical methods to detect these within-subjects differences in response processes. In psychological and educational measurement, the dominant source of information are the item responses themselves, which indicate the accuracy of the underlying response process. In this article, we will additionally focus on the item response times as a valuable additional source of information concerning the response process as they indicate the amount of time it took for the response processes to be executed (Luce, 1986). That is, everything else being equal, a systematic difference in response time suggests a difference in the underlying response process.

Various psychometric modeling approaches based on mixture modeling have been proposed that—in addition to the item responses—use the response times to identify within-subjects differences in response processes (Molenaar, Oberski, Vermunt, & De Boeck, 2016; Schnipke & Scrams, 1997; Wang & Xu, 2015; Wang, Xu, & Shang, 2018). However, although valuable, the existing mixture models are associated with at least one of the following three challenges: (1) A parametric distribution is assumed for the response times that—if violated—may bias the results; (2) the response processes are assumed to result in equal variances (homoscedasticity) in the response times, whereas some processes may produce more variability than others (heteroscedasticity; e.g., fast guessing is commonly associated with less variance than the regular response process); and (3) the different response processes are modeled as independent latent variables, whereas they may be related (e.g., after a guess, a subject may be more likely to guess on the next item).

Challenges 1, 2, and 3 have all been studied separately. That is, Challenge 1 has been addressed by Molenaar, Bolsinova, and Vermunt (2018), who proposed a mixture modeling approach based on the categorized response times to avoid assumptions about the specific parametric shape of the response time distribution. The approach was demonstrated to perform better than a parametric approach based on the log-normal response time distribution if the observed response time distribution departs from log-normality. In addition, Challenge 2 has been addressed by Wang and Xu (2015) and Wang et al. (2018), who proposed a model for two response processes, fast guessing and a regular solution process, in which the processes were heteroscedastic, that is, associated with differences in the underlying response time variance. Finally, Challenge 3 has been addressed by Molenaar et al. (2016), who modeled the possible relation between the response processes underlying two subsequent items using a time homogeneous hidden Markov process of order one.

Although the three challenges above have been addressed separately, in practice they may occur simultaneously. In the present article, we therefore propose a heteroscedastic hidden Markov mixture model for responses and categorized response times in which we explicitly address Challenges 1, 2, and 3 in a joint model. That is, we combine the categorized response time approach of Molenaar et al. (2018), the heteroscedastic response processes approach by Wang and Xu (2015) and Wang et al. (2018), and the Markov process approach of Molenaar et al. (2016) in a single model. The outline is as follows: First, the full model is derived and tested in a simulation study to investigate parameter recovery and the resolution to distinguish between different special cases. Next, the model is applied to a real dataset to demonstrate its use in practice.

## The general mixture framework

### A joint modeling approach

Within traditional item response theory models, it is assumed either that the item responses to psychometric tests are the results of a single response process (e.g., an information accumulation process; see Tuerlinckx & De Boeck, 2005; van der Maas, Molenaar, Maris, Kievit, & Borsboom, 2011) or that the response processes are homogeneous (e.g., multiple processes underlie the scores of an arithmetic test, such as subtraction and addition, but these processes are homogeneous in the sense that, statistically, they are commonly unidimensional). As a result, between-subjects differences in the accuracy of these response processes can be modeled by posing a latent ability variable, *θ*_*p*_, to underlie the item responses of respondent *p* = 1, . . . , *N* to a test. Similarly, individual differences in the speed with which these processes are executed can be captured by posing a latent speed variable, *τ*_*p*_, to underlie the response times to a test.

A joint psychometric model for responses and response times was proposed by van der Linden (2007). In this model, commonly referred to as “the hierarchical model,” the joint density of the responses, *x*_*pi*_, and the response times, *t*_*pi*_, of respondent *p* on item *i* = 1, . . . , *n*, conditional on *θ*_*p*_ and *τ*_*p*_ is denoted by *d*(*x*_*pi*_, *t*_*pi*_| *θ*_*p*_, *τ*_*p*_) = *f*(*x*_*pi*_, *t*_*pi*_| *θ*_*p*_, *τ*_*p*_). By assuming that the responses and response times are independent conditional on *θ*_*p*_ and *τ*_*p*_ (see, e.g., van der Linden, 2007; van der Linden & Glas, 2010), this conditional density can be factored into a separate response part, and a separate response time part, that is,

1 $$ f\ \left({x}_{pi},{t}_{pi}\ |\ {\theta}_p,{\tau}_p\right)=g\left({x}_{pi}\ |\ {\theta}_p\right)\times h\left({t}_{pi}\ |\ {\tau}_p\right), $$

where *g*(.) denotes the conditional probability mass function of the responses, and *h*(.) denotes the conditional density function of the response times.

**Fig. 1:**
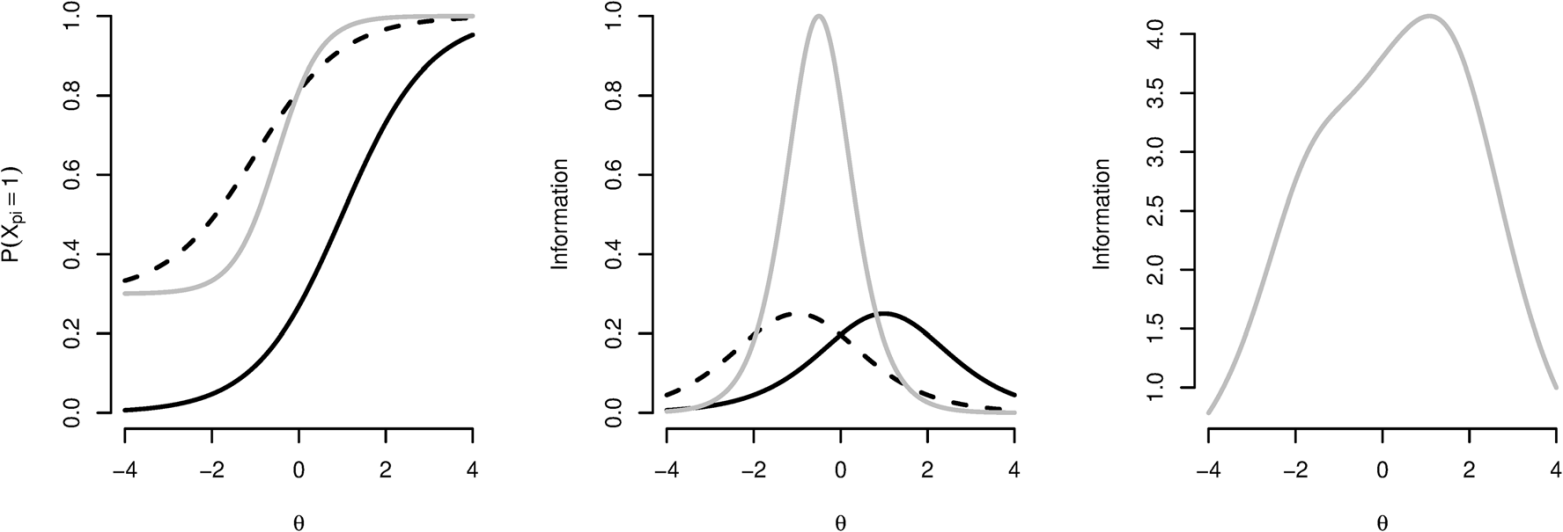
(Left) Probabilities of a correct response as a function of *θ*_*p*_, *P*(*X*_*p*i_ = 1 | *θ*_*p*_), for different parameter configurations. (Middle) The resulting item information as a function of *θ*_*p*_. Solid black line: *α*_*i*_ = 1, *β*_*i*_ = – 1, *γ*_*i*_ = 0; dashed black line: *α*_*i*_ = 1, *β*_*i*_ = 1, *γ*_*i*_ = 0; gray line: *α*_*i*_ = 2, *β*_*i*_ = 1, *γ*_*i*_ = .3. (Right) Test information as a function of *θ*_*p*_ for 25 example items (*β*_*i*_s are between – 3 and 3, *α*_*i*_s are between 0.5 and 1.5, and *γ*_*i*_ = 0)

Because psychometric test items commonly differ in the properties with which they measure the underlying processes, a model is specified for *g*(*x*_*pi*_ | *θ*_*p*_) and *h*(*t*_*pi*_ | *τ*_*p*_) in order to separate item effects and respondent effects on the responses and response times, respectively (e.g., some items are more difficulty and some respondents are faster). For instance, the three-parameter logistic item response theory model is given by

2 $$ g\left({x}_{pi}|{\theta}_p\right)=P{\left({x}_{pi}=1|{\theta}_p\right)}^{x_{pi}}{\left[1-P\left({x}_{pi}=1|{\theta}_p\right)\right]}^{1-{x}_{pi}}, $$

with the probability of a correct response given by

3 $$ P\left({x}_{pi}=1|{\theta}_p\right)={\gamma}_i+\left(1-{\gamma}_i\right)\omega \left({\alpha}_i{\theta}_p+{\beta}_i\right), $$

where *ω*(.) is a logistic or normal ogive function, and *γ*_*i*_, *α*_*i*_, and *β*_*i*_ are the item parameters. Specifically, *γ*_*i*_ is a lower-asymptote parameter that accounts for correct responses due to guessing, *α*_*i*_ is a discrimination parameter that accounts for the degree to which the item captures differences in *θ*_*p*_, and *β*_*i*_ is an easiness parameter that accounts for the proportion correct of the item. In Fig. 1 (left) is illustrated, for three example items, how these parameters affect the probability of a correct response, *P*(*x*_*pi*_ = 1 | *θ*_*p*_) in Eq. 3. Important for the assessment of between-subjects differences in the latent ability variable is the concept of “information.” That is, depending on the measurement properties of the item, an item can be more informative about *θ*_*p*_ for specific levels on the *θ*_*p*_ range. Similarly, the test as a whole does not necessarily provide an equal amount of information for each level of *θ*_*p*_. See Fig. 1 (middle) for the item information as a function of *θ*_*p*_ for the three example items from Fig. 1 (left). See Fig. 1 (right) for the test information as a function of *θ*_*p*_ for an example test of 25 items.

For the response times, similar approaches exist that separate between the latent speed variable, *τ*_*p*_, and the measurement properties of the response time variables. For instance, the log-normal model is given by

4 $$ h\left({t}_{pi}|{\tau}_p\right)=\frac{1}{\sigma_i{t}_{pi}\ }\varphi \left\{\frac{\ln \left({\mathrm{t}}_{\mathrm{pi}}\right)-\left({\nu}_i-{\lambda}_i\times {\tau}_p\right)}{\sigma_i}\right\}, $$

where *φ*(.) is the standard normal distribution function and *v*_*i*_, *λ*_*i*_, and *σ*_*i*_ are the item parameters. Specifically, *ν*_*i*_ is an intercept that accounts for the time intensity of the item (i.e., some items require more time irrespective of the difficulty, because of, for instance, a large text that has to read), *λ*_*i*_ is a factor loading that accounts for the degree with which the item captures differences in *τ*_*p*_, and *σ*_*i*_ is the standard deviation of the residual, which contains measurement error and misfit. As for the responses, the model has implications for the information about *τ*_*p*_ in the response times. That is, the information is constant over the *τ*_*p*_ range and only depends on *λ*_*i*_ and *σ*_*i*_ (see Mellenbergh, 1994).

### A mixture joint modeling approach

The general idea of the mixture approach by Schnipke and Scrams (1997), Wang and Xu (2015), Wang et al. (2018), and Molenaar et al. (2016) is to model within-subjects differences in response processes by extending the joint model above to include item-specific latent class variables, *ζ*_*pi*_, with two states *c* = 0, 1 to underlie the responses and response times of item *i*. The two states either correspond to a discrete difference in two qualitative response processes that produce heterogeneity in the data (e.g., memory retrieval and logical reasoning) or the two states correspond to two statistical states that capture heterogeneity in the data that is due to discrete differences in multiple response processes (e.g., multiple solution strategies) or due to continuous differences in one or more response processes (e.g., motivation or fatigue).

If the response processes are indeed heterogeneous, the measurement properties of *θ*_*p*_ and *τ*_*p*_ will be different across states. Therefore, in the general mixture framework, the joint conditional density of the responses, *x*_*pi*_, and the response times, *t*_*pi*_, is a mixture of the joint conditional densities of *x*_*pi*_ and *t*_*pi*_ within the two states, that is

5 $$ d\left({x}_{pi},{t}_{pi}|{\theta}_p,{\tau}_p\right)={\sum}_{\zeta_{pi}=0}^1P\left({\zeta}_{pi}\right){f}_c\left({x}_{pi},{t}_{pi}|{\theta}_p,{\tau}_p,{\zeta}_{pi}\right) $$

where *f*_*c*_(.) is the joint density function within state *ζ*_*pi*_ = *c*, and *P*(*ζ*_*pi*_) is the state probability. Within each state, the responses and response times are still assumed to be independent conditional on *θ*_*p*_ and *τ*_*p*_, that is

6 $$ {f}_c\left({x}_{pi},{t}_{pi}|{\theta}_p,{\tau}_p,{\zeta}_{pi}\right)={g}_c\ \left({x}_{pi}|{\theta}_p,{\zeta}_{pi}\right)\times {h}_c\ \left({t}_{pi}\ |\ {\tau}_p,{\zeta}_{pi}\right), $$

where *g*_*c*_(.) denotes the conditional probability mass function of the responses in state *c* and *h*_*c*_(.) denotes the conditional density function of the response times in state *c*. In the general mixture framework, for the within-state response time density, the log-normal linear model from Eq. 4 is used as follows

7 $$ {h}_c\left({t}_{pi}|{\tau}_p,{\zeta}_{pi}=c\right)=\frac{1}{\sigma_{ci}{t}_{pi}\ }\varphi \left\{\frac{\ln \left({\mathrm{t}}_{\mathrm{pi}}\right)-\left({\nu}_{ci}-{\lambda}_{ci}\times {\tau}_p\right)}{\sigma_{ci}}\right\}, $$

where the item parameters are allowed to differ across states as indicated by index c. For the responses, the three-parameter item response theory model from Eq. 3 is used:

8 $$ {g}_c\left({x}_{pi}|{\theta}_p,{\zeta}_{pi}=c\right)=P{\left({x}_{pi}=1|{\theta}_p,{\zeta}_{pi}=c\right)}^{x_{pi}}{\left[1-P\left({x}_{pi}=1|{\theta}_p,{\zeta}_{pi}=c\right)\right]}^{1-{x}_{pi}}, $$

with

9 $$ P\left({x}_{pi}=1|{\theta}_p,{\zeta}_{pi}=c\right)={\gamma}_{ci}+\left(1-{\gamma}_{ci}\right)\omega \left({\alpha}_{ci}{\theta}_{pi}+{\beta}_{ci}\right), $$

where the item parameters are again allowed to differ across states. The framework given by Eqs. 5, 6, 7, 8, and 9 is very general, in the sense that it includes many parameters that are not identified simultaneously and that are yet difficult to interpret. However, various special cases within this general framework have been considered in the literature. See Table 1 for the exact restrictions needed to arrive at these special cases.^1^

**Table 1:** Parameter restrictions in the general mixture framework necessary to obtain special cases from the literature

			Response Times			Responses		
Model	References	*c*	*ν*_*ci*_	*λ*_*ci*_	*σ*_*ci*_	*γ*_*ci*_	*α*_*ci*_	*β*_*ci*_
Hierarchical model (baseline)	van der Linden (2007)	0	*ν*_0 *i*_	1	*σ*_0 *i*_	*γ*_0 *i*_	*α*_0 *i*_	*β*_0 *i*_
		1	*–*	*–*	*–*	*–*	*–*	*–*
Standard mixture model	Schnipke and Scrams (1997)	0	*ν*_0 *i*_	0	*σ*_0 *i*_	*–*	*–*	*–*
		1	*ν*_1 *i*_	0	*σ*_1 *i*_	*–*	*–*	*–*
Common-guessing mixture model	Schnipke and Scrams (1997)	0	*ν*_0_	0	*σ*_0_	*–*	*–*	*–*
		1	*ν*_1 *i*_	0	*σ*_1 *i*_	*–*	*–*	*–*
Mixture hierarchical model	Wang and Xu (2015); Wang et al. (2018)	0	*ν*_0_	0	*σ*_0_	0	0	*β*_0 *i*_
		1	*ν*_1 *i*_	1	*σ*_1 *i*_	*γ*_*ci*_	*α*_1 *i*_	*β*_1 *i*_
Independent-states mixture model	Molenaar et al. (2016)	0	*ν*_0 *i*_	1	*σ*_*i*_	0	*α*_0 *i*_	*β*_0 *i*_
		1	*ν*_0 *i*_ *+δ*_1_	1	*σ*_*i*_	0	*α*_1 *i*_	*β*_1 *i*_

“–” denotes that this part of the general model is omitted (i.e., for the hierarchical model by van der Linden, 2007, there is no Class 1 in the model, and for the models by Schnipke & Scrams, 1997, there is no measurement model for the responses)

From the table it can be seen that the first model, the hierarchical model by van der Linden (2007) discussed above, arises by specifying a log-normal model with λ_0*i*_ = 1 for the response times, and a three-parameter model for the responses in state 0 and leaving state 1 empty. Because this model assumes a single state only, it corresponds to a single-process model or homogeneous process model that can be used as a baseline in drawing inferences about within-subjects differences in response processes in the data. Note that the factor loadings are constrained to be equal to 1 in the single-state model and in all other models that include *τ*_*p*_, which is an essentially tau-equivalent factor model (Lord & Novick, 1968). This assumption has been relaxed in the hierarchical model by, for instance Fox, Klein Entink, and van der Linden (2007) and Molenaar, Tuerlinckx, and van der Maas (2015).

The next two models in Table 1 are by Schnipke and Scrams (1997). These models consider response times only. As can be seen, both models do not include a latent speed variable as λ_ci_ = 0 in both states. In the standard mixture model, the intercept and variance are estimated for each item in both states. In the common-guessing mixture model, the intercepts and variances in Class 0 (the guessing class) are restricted to be equal across items. Although these models by Schnipke and Scrams are not latent variable models, to our knowledge, these models have been the first to include a within-subjects mixture component for response times. In addition, the idea of common-guessing has been adopted by Wang and Xu (2015) and Wang et al. (2018), who proposed a common-guessing latent-variable model for both responses and response times. As can be seen in Table 1, the response time model includes a latent speed variable in state 1 (i.e., *λ*_1*i*_ = 1) with item-specific intercepts and residual variances, and a common intercept and residual variance in state 0, but without a latent speed variable. In addition, the response model includes a three-parameter latent-variable model for the responses in state 1 and a fast-guessing parameter *β*_0*i*_ in state 0 without a latent variable. Finally, Molenaar et al. (2016) proposed a model with a latent speed variable in both states (i.e., *λ*_0*i*_ = 1 and *λ*_1*i*_ = 1), in which the item-specific intercepts in state 1 are equal to the intercepts of state 0 shifted by a common scalar, *δ*_1_. In addition, the residual standard deviation is assumed to be equal across states (*σ*_*ci*_ = *σ*_*i*_). For the responses, a two-parameter model is used in both states (*γ*_*ci*_ = 0).

## Challenges and a possible solution

### The response time distribution

The mixture approaches discussed above are all associated with one of the following challenges. First, the approaches all assume a log-normal distribution for the response times within the states. As has been argued by Vermunt (2011) for standard mixture models, and demonstrated by Bauer and Curran (2003) for growth mixture models and by Molenaar et al. (2018) for the independent states mixture model in Table 1, violations of the assumed within-states distribution may result in (1) spurious states—that is, states that are not actually in the data but appear as a significant source of variation in the modeling to capture the misfit in the data distribution—and (2) biased true states—that is, differences between true states (that are actually in the data) may seem smaller or larger depending on the source of the misfit in the data distribution (e.g., positive skew or negative skew, truncation, etc.).

In principle, this challenge can be solved by specifying a more appropriate response time distribution within each state. However, commonly there is no theory about the response time distribution within each state. In addition, inferring the within-state response time distribution from the data is difficult, because only the observed distribution of the response times is available, which cannot straightforwardly be used to make inferences about the parametric form of the within-state distribution as the observed response time distribution will depart from the within-state distribution by definition. Kuipers, Visser, and Molenaar (2018) proposed a test on log-normality of the within-state response time distribution. However, if the log-normality assumption fails, the above mixture models are not suitable for the data.

As a solution, Molenaar et al. (2018) proposed to categorize the continuous response times so that the resulting response time distribution could be better captured using category-specific threshold parameters. Specifically, Molenaar et al. (2018) proposed to replace the log-normal linear model above by a partial-credit model (Masters, 1982), which is an adjacent-category model for ordered categories, or any other model for ordered categories (e.g., the graded response model [Samejima, 1969], which is a cumulative probability model). With respect to the categorization of the response times, Molenaar et al. (2018) proposed to use an item-wise categorization procedure using the observed percentiles. For five or seven categories, this approach worked well in terms of both parameter recovery and power.

### Dependency between the states

In the general model in Eq. 5, it is assumed that the latent class variables underlying the items, *ζ*_*pi*_, are independent. However, various examples show why the *ζ*_*pi*_ variables can be dependent. First, if a respondent guesses on one item, it may be more likely that this respondent will also guess on the next item. A similar example includes response strategies in general. That is, if multiple solution strategies are possible that differ in their efficiency, using an efficient solution strategy on one item will probably increase the probability that this strategy will also be used on the next item. Another example includes posterror slowing (Rabbitt, 1979), which refers to the phenomenon that respondents, who know (or think) that they made an error on a given item, slow down on the next item resulting in a dependency between subsequent *ζ*_*pi*_s.

Within the general mixture framework in Eq. 5, Molenaar et al. (2016) accounted for a possible dependency of the item specific latent class variables of item *i*, *ζ*_*pi*_, on the item specific latent class variables of item *i* – 1, *ζ*_*p*(*i*–1)_. That is, in a model for continuous log-normal response times, the assumption of independent *ζ*_*pi*_ was relaxed by introducing a first-order Markov structure (e.g., MacDonald & Zucchini, 1997) on *ζ*_*pi*_. Molenaar et al. (2016) showed that the presence of a Markov structure in the data can successfully be detected using fit indices BIC, CAIC, AIC with a triple penalty (AIC3), and the sample-size-adjusted BIC (saBIC). The conventional AIC (which uses a double penalty term) was associated with an increased false positive rate.

### Heteroscedasticity between the states

The categorized response time model and the Markov structure thus provide a solution to the spurious-state and independency challenges of the general framework in Eq. 5. However, contrary to Wang and Xu (2015), Wang et al. (2018), and Schnipke and Scrams (1997), both models assume that the within-state response time variance is homoscedastic (equal across states). In the Markov mixture model, this assumption is explicit, as *σ*_0*i*_ = *σ*_1*i*_ in the model by Molenaar et al. (2016). In the categorized response time model it is less explicit, since traditional item response theory models do not have a variance parameter. However, the same thresholds are applied in both states to categorize the response times (since the marginal response time distribution is categorized and not the within-state response time distribution, because this distribution is unknown). Therefore, heteroscedasticity across states will not be detected and will bias the results, as we will demonstrate in the simulation study below.

### Proposed model

In this article, we thus propose a model that combines the categorized response time model by Molenaar et al. (2018), the Markov model by Molenaar et al. (2016), and the heteroscedastic state model by Wang and Xu (2015), Wang et al. (2018), and Schnipke and Scrams (1997) into a single model. First, to be able to accommodate the general model in Eq. 5 to include a Markov dependence among *ζ*_*pi*_, we need to consider the conditional density of the full vector of responses, ***x***_***p***_ = [*x*_*p*1_, . . . , *x*_*pn*_], and the full vector of categorized response times, ***t***_***p***_***'*=** [*t*_*p*1_*'*, . . . , *t*_*pn*_*'*], where *t*_*pi*_*'* denotes the categorized response times, *t*_*pi*_*'* = 0, 1, . . . , *T*–1. Next, Eqs. 5 and 6 change into

10 $$ d\left({x}_p,{t}_p^{\hbox{'}}|{\theta}_p,{\tau}_p\right)={\sum}_{\zeta_{p1}=0}^1\dots {\sum}_{\zeta_{pn}=0}^1P\left({\zeta}_{p1}\right){\prod}_{i=2}^nP\left({\zeta}_{pi}|{\zeta}_{p\left(i-1\right)}\right){\prod}_{i=1}^n{g}_c\left({x}_{pi}|{\theta}_p,{\zeta}_{pi}\right){h}_c\left({t}_{pi}^{\hbox{'}}|{\tau}_p,{\zeta}_{pi}\right) $$

where *P*(*ζ*_*p*1_ = 1) = *π*_1_ is the initial state parameter, and *P*(*ζ*_*pi*_ = 1| *ζ*_*p*(*i* − 1)_ = 0) = *π*_1|0_ and *P*(*ζ*_*pi*_ = 1| *ζ*_*p*(*i* − 1)_ = 1) = *π*_1|1_ are the transition parameters. Note that *P*(*ζ*_*p*1_ = 0), *P*(*ζ*_*pi*_ = 0| *ζ*_*p*(*i* − 1)_ = 0), and *P*(*ζ*_*pi*_ = 0| *ζ*_*p*(*i* − 1)_ = 1) can be calculated from these parameters. In addition, we assume homogeneity of the Markov structure over items. That is, the transition probabilities are invariant over all items, *P*(*ζ*_*pi*_|*ζ*_*p*(*i*–1)_) = *P*(*ζ*_*pj*_|*ζ*_*p*(*j*–1)_) for all *i* and all *j* = 1, . . . , *n*. This assumption is common in Markov modeling (e.g., Bacci, Pandolfi, & Pennoni, 2014; Gudicha, Schmittmann, & Vermunt, 2016; Zucchini, MacDonald, & Langrock, 2016, p. 15). Besides being common practice, here, we also assume time homogeneity of the Markov structure to prevent the model from becoming too complex. Including a time non-homogeneous Markov structure would result in two additional parameters for each item [probability of remaining in a class, *P*(*ζ*_*pi*_ = 1 | *ζ*_*p*(*i*–1)_ = 1), and the probability of switching classes, *P*(*ζ*_*pi*_ = 1 | *ζ*_*p*(*i*–1)_ = 0)] which makes the model very complex. Given that the model from Molenaar et al. (2018) already includes four parameters for each item response variable and *T* – 1 response time category parameters, we did not consider such an extension of the homogeneous Markov structure into a nonhomogeneous Markov structure. However, this extension is straightforward (i.e., in the syntax to fit the model in the Appendix, which will be explained later, we indicate how to drop the time homogeneity assumption). In addition, the assumption of time homogeneity can be statistically tested (see, e.g., Tan & Yılmaz, 2002).

Next, for the conditional probability function of the categorized response times, *h*_*c*_(.), we use the partial credit model subject to heteroscedasticity (Hedeker, Berbaum, & Mermelstein, 2006), as follows:

11 $$ {\displaystyle \begin{array}{l}{h}_c\left({t}_{pi}^{\hbox{'}}=t|{\tau}_p,{\zeta}_{pi}=c\right)=P\left({t}_{pi}^{\hbox{'}}=t|{\tau}_p,{\zeta}_{pi}=c\right)=\frac{\exp \left({\sum}_{z=0}^t\frac{\upnu_{\mathrm{iz}}-{\delta}_c-{\lambda}_i{\tau}_p}{\sigma_c}\right)}{\sum_{j=0}^{T-1}\exp \left({\sum}_{z=0}^j\frac{\upnu_{\mathrm{iz}}-{\delta}_c-{\lambda}_i{\tau}_p}{\sigma_c}\right)},\\ {}\mathrm{with}\;{\delta}_1,{\sigma}_c>0,\end{array}} $$

where *ν*_*it*_ denotes the threshold of response time category *t* on item *i* with ν_i0_ arbitrarily set to 0. In Eq. 11, we assume the intercepts and loadings to be invariant across states but we model a scale and location difference between the states using, respectively, *δ*_*c*_ and *σ*_*c*_. That is, if *δ*_0_ = 0 for identification purposes, *δ*_1_ accounts for a location shift of the thresholds in state 1 as compared to the thresholds in state 0. This reflects that the average raw response times are different between the states. As *δ*_1_ > 0, the responses in Class 1 are on average faster than the responses in Class 0. Parameter *σ*_*c*_ accounts for a scale difference in state 1 as compared to state 0, which is due to the raw response times being more variable in one state than in the other (heteroscedasticity). Note that in the traditional partial-credit model with only one state *σ*_*c*_ = *σ* is only identified if two thresholds are fixed (Mehta, Neale, & Flay, 2004). However, here, if *σ*_0_ = 1 for identification purposes, parameter *σ*_1_ is identified and represents the ratio between the residual standard deviations in the two states. Thus, in the case of homoscedasticity *σ*_0_ = *σ*_1_ = 1. In the case of heteroscedasticity, *σ*_1_ > 1, denotes more variability in state 1 and *σ*_1_ < 1 denotes more variability in state 0. In the model for categorized response times in Eq. 11, differences in variability between items (i.e., differences in *σ*_*ci*_ across *i* in the continuous response time model in Eq. 7) are captured in the thresholds, *ν*_*i*_ and the factor loadings, *λ*_*i*_. Differences in variability between classes are captured by *σ*_*c*_.

Finally, for the conditional probability mass function of the responses within each state, *g*_*c*_(*x*_*pi*_| *θ*_*p*_, *ζ*_*pi*_ = *c*), we use Eq. 8 with a two-parameter model for *P*(*x*_*pi*_ = 1 | *θ*_*p*_, *ζ*_*pi*_ = *c*), that is,

12 $$ P\left({x}_{pi}=1|{\theta}_p,{\zeta}_{pi}=c\right)=\omega \left({\alpha}_{ci}{\theta}_{pi}+{\beta}_{ci}\right). $$

Note that, contrary to Wang and Xu (2015) and Wang et al. (2018), we follow Molenaar et al. (2018; Molenaar et al., 2016) and use a two-parameter model for the responses (see also Table 1). Our main reason is that we want to operate in a generalized linear modeling framework that does not include the three-parameter model as a special case.^2^ Using a three-parameter model would increase our model complexity, resulting in a potentially poorly identified model. Within the generalized linear modeling framework, we are sure that the model is identified and can be estimated properly. In addition, our modeling interest is mainly in detecting possible differences in item discrimination and item easiness across the different states (suggesting different response processes). However, extending the present model to a three-parameter model would be possible in principle

The model given by Eq. 10, with *h*_*c*_(.) given by Eq. 11, *g*(.) given by Eq. 8, and *P*(*x*_*pi*_ = 1|*θ*_*p*_, *ζ*_*pi*_ = *c*) given by Eq. 12, constitutes the *heteroscedastic hidden Markov mixture model*. If we assume a bivariate standard normal distribution for *τ*_*p*_ and *θ*_*p*_ with correlation *ρ*, and if ***η*** denotes the vector of free parameters in the model (i.e., *α*_0*i*_, *α*_1*i*_, *β*_0*i*_, *β*_1*i*_, and *λ*_*i*_ for all *i*, *ν*_*it*_ for all *i* and for *t* = 1, . . . , *T* – 1, and *δ*_1_, *σ*_1_, *π*_1_, *π*_0|1_, and *ρ*), then the resulting full marginal log-likelihood function of the model is given by

13 $$ \ell \left(\boldsymbol{\upeta} \right)=\mathit{\ln}\int \underset{-\infty }{\overset{\infty }{\int }}\sum \limits_{\zeta_{p1}=0}^1\dots \sum \limits_{\zeta_{pn}=0}^1P\left({\zeta}_{p1}\right)\prod \limits_{i=2}^nP\left({\zeta}_{pi}|{\zeta}_{p\left(i-1\right)}\right)\prod \limits_{i=1}^n{g}_c\left({x}_{pi}|{\theta}_p,{\zeta}_{pi}\right){h}_c\left(\underset{pi}{\overset{\hbox{'}}{t}}|{\tau}_p,{\zeta}_{pi}\right)k\left({\tau}_p,{\theta}_p\right) d\theta d\tau, $$

where *k*(.) is a bivariate standard normal distribution with correlation *ρ*.

We focus on five instances of the general model above:

1. *Baseline:* A baseline model with one state (see Table 1).
2. *Heteroscedastic Markov states:* The full model with a Markov structure on the latent class variables and heteroscedastic states.
3. *Homoscedastic Markov states:* A model with a Markov structure on the latent class variables and homoscedastic states.
4. *Heteroscedastic independent states:* A model with independent latent class variables and heteroscedastic states
5. *Homoscedastic independent states:* A model with independent latent class variables and homoscedastic states

In all models, we use categorized response times. In the simulation study below, we investigate the viability of the general model in terms of parameter recovery and the resolution to distinguish between the different models above in responses and categorized response time data.

### Categorization of response times

The models proposed require categorization of the continuous response times. Because the results potentially depend on the exact categorization scheme, categorization should be done with care. In the partial credit model above, the adjacent categories logit in the baseline model (i.e., *δ*_*c*_ = 0 and *σ*_*c*_ = 1 for all *c*) is given by

$$ \log \left\{\frac{h_c\left({t}_{pi}^{\prime }=t|{\tau}_p\right)}{h_c\left({t}_{pi}^{\prime }=t-1|{\tau}_p\right)}\right\}={\nu}_{it}-{\lambda}_i{\tau}_p. $$

**Fig. 2:**
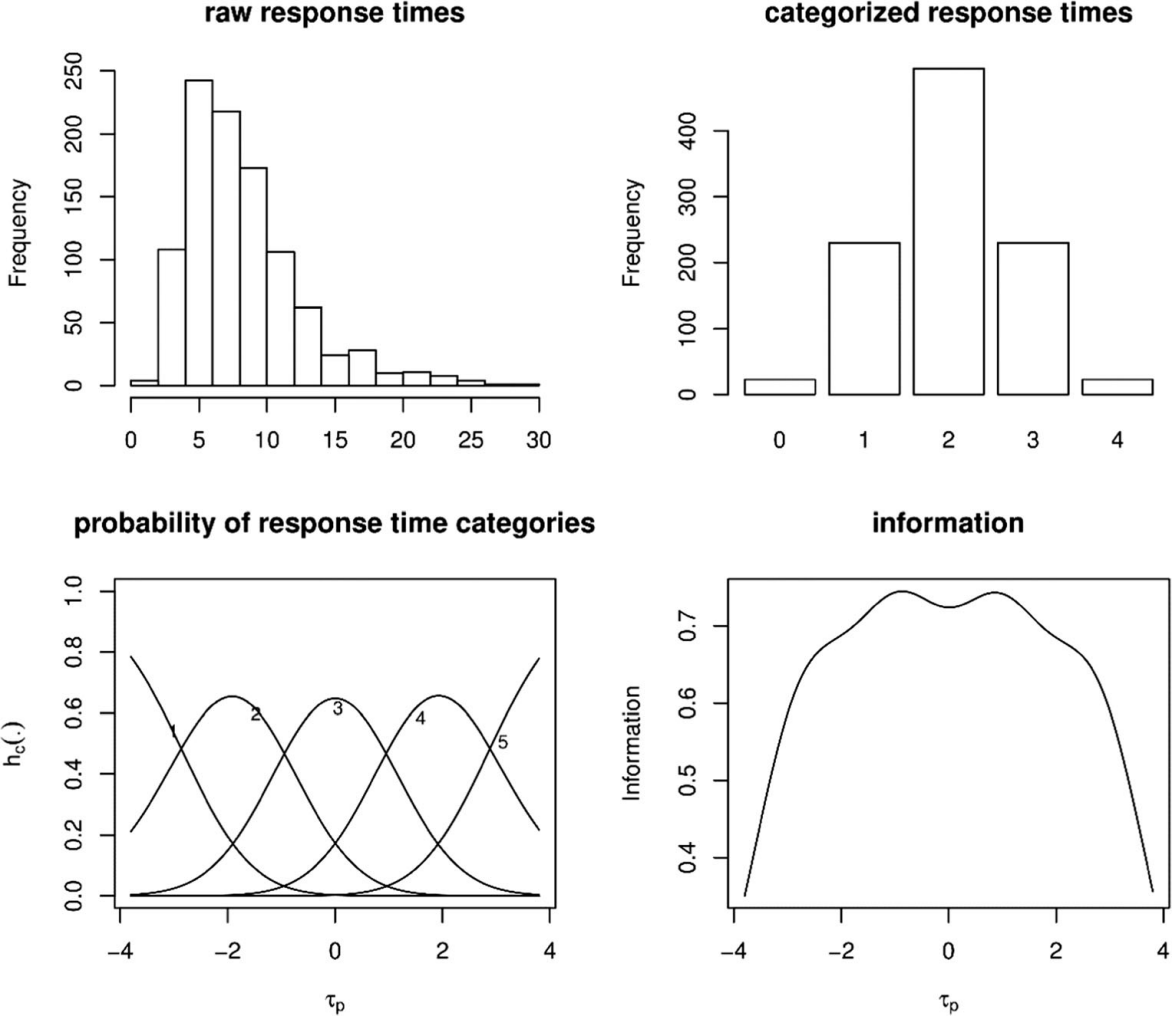
Illustration of the categorization procedure, based on percentiles derived from a normal distribution (2nd, 25th, 75th, and 98th percentiles). (Top left) Histogram of the raw response times. (Top right) Bar plot of the categorized response times. (Bottom left) Conditional probabilities of each response time category. (Bottom right) Information in the categorized response times as a function of the latent speed variable *τ*_p_

**Fig. 3:**
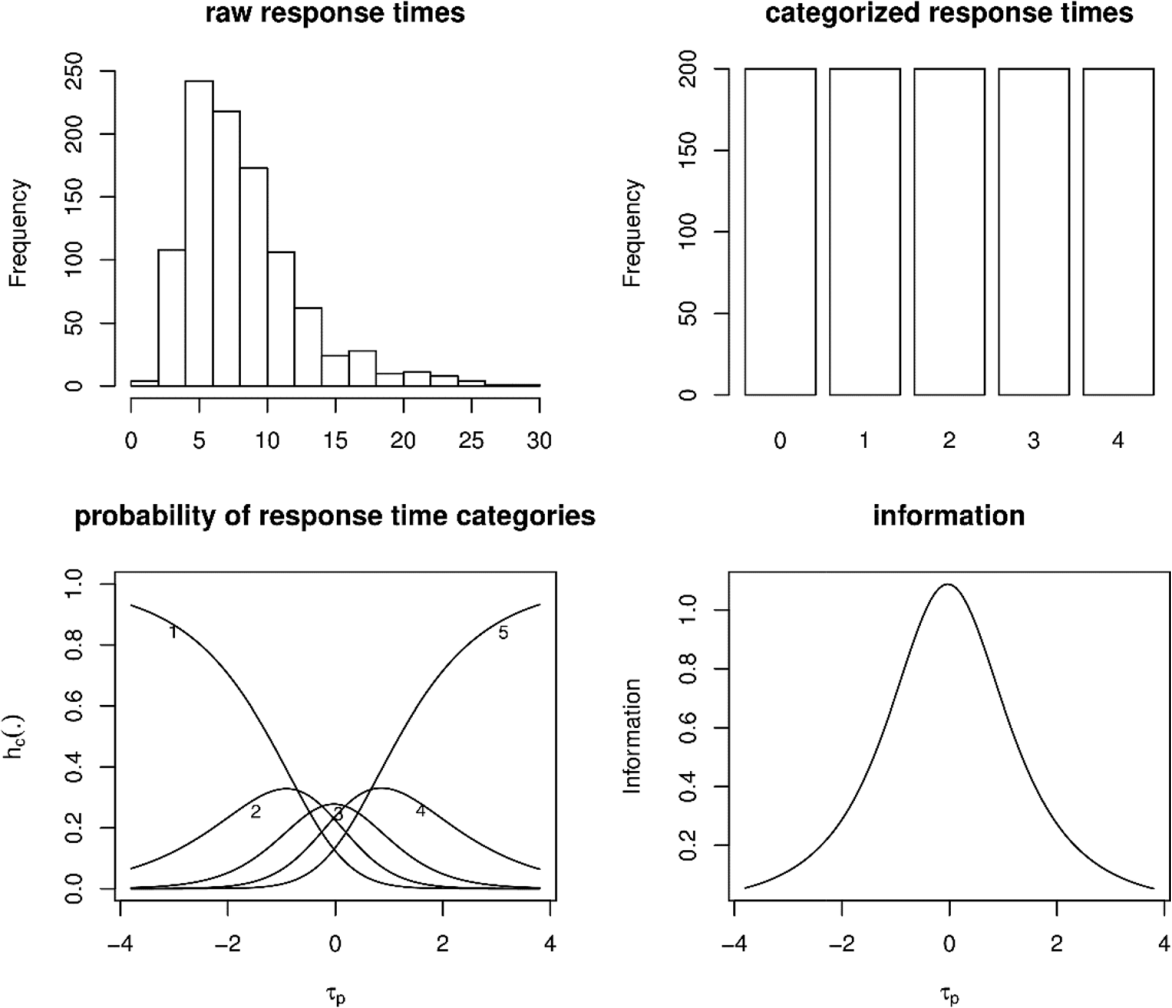
Illustration of the categorization procedure based on equidistant percentiles (the 20th, 40th, 60th, and 80th percentiles). (Top left) Histogram of the raw response times. (Top right) Bar plot of the categorized response times. (Bottom left) Conditional probabilities of each response time category. (Bottom right) Information in the categorized response times as a function of the latent speed variable *τ*_p_

In this equation, the threshold parameter *ν*_*it*_ is directly influenced by the cut-off values at which the continuous response times are categorized. In principle, this is not a problem, as the other parameters are relatively unaffected by the exact choice of the cutoff values. However, this choice does affect the power to detect differences between states. Therefore, the cutoff values should be chosen in an optimal way. Here we propose to categorize the continuous response times in such a way that the adjacent categories logits show large, but, constant differences across categories. This will result in thresholds parameters *ν*_*it*_ that are equidistant and well spread over the *τ*_*p*_ range so that the information about *τ*_*p*_ in the categorized response times is approximately constant over *τ*_*p*_ (at least in the interval – 3, 3). A possible way to accomplish this is to choose the cutoff values on basis of equally spaced values in a symmetrical distribution (e.g., logistic or normal distribution). Here we use – 2, *–* 2*/*3, 2*/*3, and 2 in a normal distribution. This corresponds to cumulative probabilities of .0228, .2525, .7475, and .9773, which are used to categorize the continuous response times (i.e., at percentiles 2.28, 25.25, 74.75, and 97.73). In Fig. 2, this procedure is illustrated for a simulated-data example. Specifically, for a single item response time variable, the figure contains a histogram of the raw response times, a bar plot of the categorized response times, a plot of the conditional probability of each response time category, and the information of the categorized response times across *τ*_*p*_. Applying the partial-credit model to data such as those in Fig. 2 will result in *ν*_*it*_ estimates that are well spread out over the *τ*_*p*_ range (at least in the – 3, 3 range), such that the information about *τ*_*p*_ is relatively constant in the range (– 3, 3). An alternative approach to categorizing the continuous response times may be to use equidistant percentiles like 20, 40, 60, and 80; however, as is illustrated in Fig. 3, such an approach will result in conditional response time category probabilities (bottom left plot) that are mainly centered around *τ*_*p*_ = 0. Applying the partial-credit model to data such as those in Fig. 3 will result in ν_it_ estimates that are close together for a given item *i*. As a result, the information about the latent speed variable, *τ*_*p*_, peaks at 0 and decreases relatively fast for values further away from 0. In the present study, we therefore consider the former approach (based on percentiles derived from a normal distribution at – 2, *–* 2*/*3, 2*/*3, and 2).

### Estimation

The models above were implemented in LatentGold (Vermunt & Magidson, 2013) and estimated using marginal maximum likelihood. We optimized the marginal log-likelihood function in Eq. 13 above by numerically integrating the double integral using ten quadrature points for each dimension. Next, we used the Baum–Welch adapted EM algorithm (Baum, Petrie, Soules, & Weiss, 1970; Welch, 2003) to obtain reasonable starting values, after which we used the Newton–Raphson algorithm to find the maximum of the likelihood function. Because this procedure is full-information, missing data in the responses or the response times do not pose a problem as long as these are missing at random (Little & Rubin, 1987). The syntax to fit the full model (heteroscedastic Markov states) is available in the Appendix.

## Simulation study

### Design

To study the viability of the proposed models, we investigated the parameter recovery of the latent state parameters *α*_*ic*_, *β*_*ic*_, *π*_1_, *π*_1|0_, and *π*_1|1_. We considered the situation in which the response time distribution departs from a log-normal distribution such that the continuous response time mixture model for the response times in Eq. 7 is unsuitable (i.e., as it will produce bias and false positives as discussed above).

**Fig. 4:**
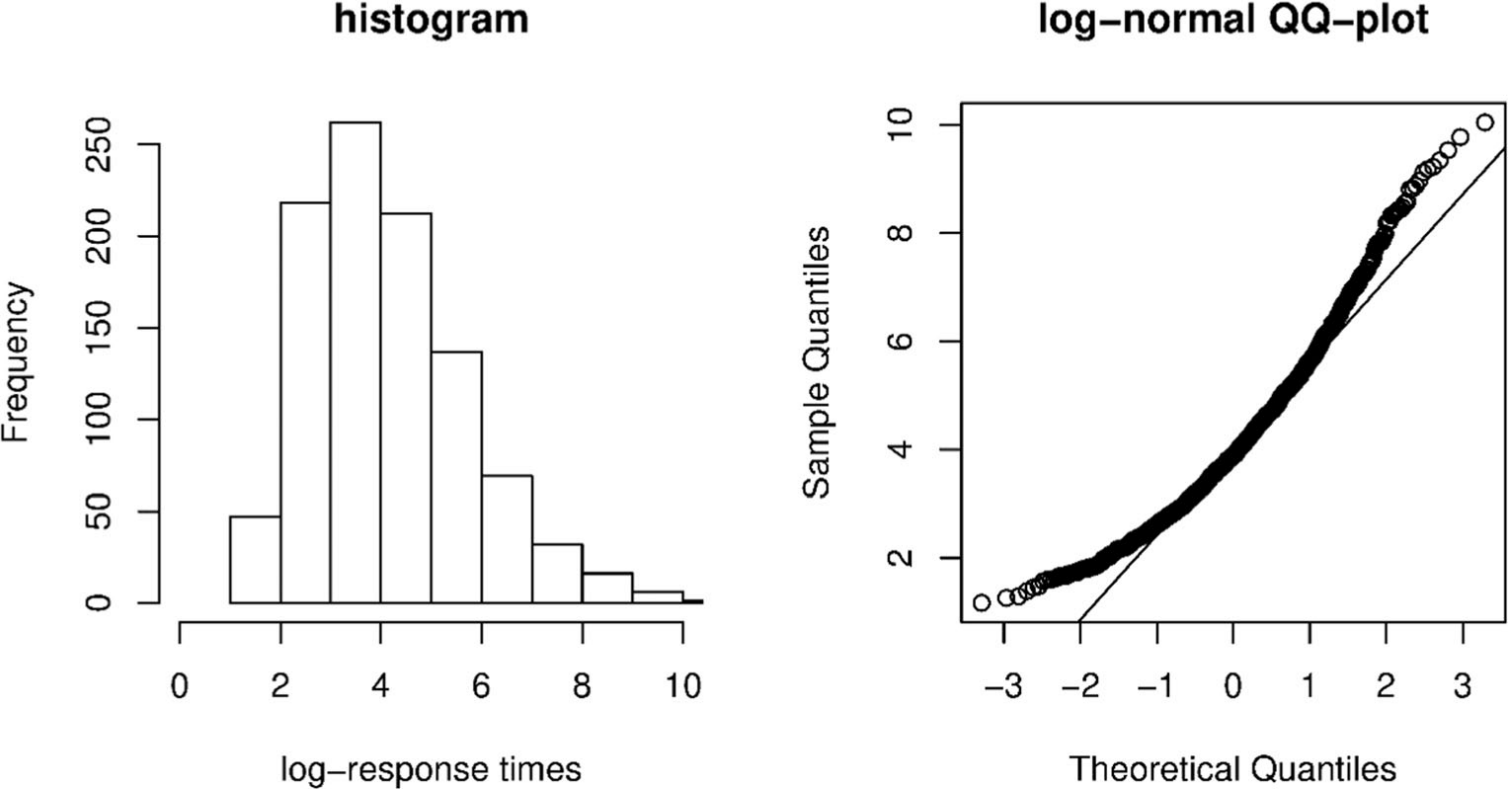
Example distribution of continuous response times in the simulation study, which depart from a log-normal distribution. These continuous response times are subsequently categorized. (Left) Histogram of the log-transformed continuous response times (which should be normal if the response times followed a log-normal distribution). (Right) Log-normal QQ-plot of the response times (which should be on the straight line if the response times followed a log-normal distribution)

The general procedure was as follows: We simulated responses and response times for 1,000 respondents on 20 items according to five scenarios that correspond to the five models above. We first simulated responses and continuous response times, after which the response times were categorized. Continuous response time data for the five scenarios were simulated according to a Box–Cox-transformed log-normal response time model that corresponds to the given scenario (e.g., for the heteroscedastic Markov states scenario, this will be a heteroscedastic Markov states model in which the partial credit model in Eq. 11 is replaced by a Box–Cox-transformed log-normal model). The Box–Cox transformation was used in order to make the response time data overly skewed, such that the response times do not follow a log-normal distribution, which invalidates models like the one in Eq. 7 discussed above. Below we discuss how we exactly simulated the responses and continuous response time data in each scenario:

- ***Heteroscedastic Markov states*** To generate data for the first scenario, we used the heteroscedastic Markov states model with a continuous log-normal response time distribution with mean *ν*_*i*_ − *δ*_*c*_ − *τ*_*p*_ and standard deviation *σ*_*c*_, which is the continuous version of Eq. 11 from the heteroscedastic Markov states model for categorized response times. For the mixture parameters, we used *π*_1_ = .666 for the initial state parameter and *π*_0|1_ = .231 and *π*_1|1_ = .769 for the transition parameters (note that these choices imply that *π*_0_ = .333, *π*_1|0_ = .231, and *π*_0|0_ = .769). These effect sizes correspond to moderately imbalanced initial state probabilities (Dias, 2006) and moderately unstable transition parameters (Bacci et al., 2014). The responses were simulated using *α*_0*i*_ = 1.5 and *α*_1*i*_ = 1 for all *i* for the discrimination parameters. For the easiness parameter, we used increasing, equally spaced values between – 2 and 0 for *β*_0*i*_ and between 0 and 2 for *β*_1*i*_. For the response times, we simulated *τ*_*p*_ with *σ*_*τ*_ = *√*0.13 and a correlation between *τ*_*p*_ and *θ*_*p*_ of .4. For the intercepts, we used *ν*_*i*_ = 2 for all *i*, *δ*_0_ = 0, and *δ*_1_ = 0.5. For the residual standard deviations, we used *σ*_0_ = *√*0.39 and *σ*_1_ = *√*0.13. These choices result in communalities of .25 in Class 0 and .5 in Class 1 on the log-scale (as we simulated log-normal data; see above). In addition, the intercept differences of 0.5 between the states were considered of medium effect size by Molenaar et al. (2018). After the log-normal response time data were simulated, we log-transformed the simulated response times resulting in normally distributed log-response times. These log-response times were subsequently transformed using the Box–Cox transformation, *ξ*(*x* + 1)^*ζ*^, with transformation parameter *ξ* = 0.3, such that the raw response times (i.e., the exponentially transformed Box–Cox log-response times) are overly skewed as compared to a log-normal distribution. As we mentioned, this makes these data unsuitable for mixture models like the one in Eq. 7, calling for our categorized response time mixture model. See Fig. 4 for an example response time distribution from the present simulation study.
- ***Homoscedastic Markov states*** In this scenario, we used the same setup and procedure as for the Heteroscedastic-Markov-States scenario but with *σ*_0_ = *σ*_1_ = *√*0.13.
- ***Heteroscedastic independent states*** In this scenario, we used the same setup and procedure as in the Heteroscedastic-Markov-States scenario but without the Markov structure on the states (i.e., *P*(*ζ*_*pi*_ = 1) = *π*_1_ for all *i*)
- ***Homoscedastic independent states*** In this scenario, we used the same setup and procedure as in the heteroscedastic independent states scenario, but with *σ*_0_ = *σ*_1_ = √0.13.
- ***Baseline*** In this scenario, we used a baseline model without mixture (i.e., only one state: *δ*_0_ = *δ*_1_ = 0, *σ*_0 =_*σ*_1_ = *σ*, *α*_0*i*_ = *α*_1*i*_ = *α*_*i*_, and *β*_0*i*_ = *β*_1*i*_ = *β*_*i*_). For the response time parameters *ν*_*i*_ and *σ*_*i*_, we used the parameters from state 0 in the homoscedastic independent states model above. For the responses we used *α*_*i*_ = 1.5 and equally spaced values between – 2 and 2 for *β*_*i*_. All other parameters were the same as in the homoscedastic independent states model above. In addition, like in the other scenarios, the response times data were transformed according to the Box–Cox transformation as explained above.

After the responses and continuous response times had been simulated, the raw response times were categorized at percentiles 2.28, 25.25, 74.75, and 97.73, resulting in five response time categories. Note that it does not make a difference whether the raw or transformed response times are categorized as the percentile scores will be the same. The percentiles that we used are obtained from a standard normal distribution at – 2, *–* 2*/*3, 2*/*3, and 2.

We used 50 replications for each data scenario. To the replications within each data scenario we fit the five models discussed above. Note that we thus did not fit the true model to the simulated data as the data were generated according to the Box–Cox-transformed log-normal model, and we fit a model for categorized response times. However, if the categorized model is viable, the latent state parameters *α*_*ic*_, *β*_*ic*_, *π*_1_, *π*_1|0_, and *π*_1|1_ should be correctly recoverable despite the response times being categorized. The recovery of the response time measurement model parameters *ν*_*it*_, *λ*_*i*_, and *σ*_*c*_ cannot be studied as they do not have a corresponding true parameter value.

For each model we considered which of the five models is the best-fitting model according to the following fit indices: the Bayesian information criterion (BIC; Schwarz, 1978), Akaike’s information criterion (AIC; Akaike, 1974), AIC3 (Bozdogan, 1993), the consistent AIC (CAIC; Bozdogan, 1987), and the sample-size-adjusted BIC (saBIC; Sclove, 1987). All these fit indices are based on the maximum marginal log-likelihood, $\ell \left(\widehat{\boldsymbol{\eta}}\right)$, where $\widehat{\boldsymbol{\eta}}$ contain the parameter values that maximize *ℓ*(**η**) from Eq. 13. That is, the general form of these fit indices is: $-2\ell \left(\widehat{\boldsymbol{\eta}}\right)+P$. The main difference between the fit indices above is the penalty term, *P*, that is used. That is,

$$ {\displaystyle \begin{array}{l}\mathrm{AIC}:P=2\times npar,\\ {}\mathrm{BIC}:P=\log (N)\times npar,\\ {}\mathrm{AIC}3:P=3\times npar,\\ {}\mathrm{CAIC}:P=2\times npar\left( npar-1\right)/\left(N- npar-1\right),\\ {}\mathrm{saBIC}:P=\log \left(\left(\mathrm{N}+2\right)/24\right)\times npar,\end{array}} $$

where *npar* denotes the number of estimated parameters in a given model. For all the fit indices it holds that a smaller value indicates a better model fit.

### Results

#### Parameter recovery

We limit our presentation of the parameter recovery results to the most complex model (heteroscedastic Markov states model) as this is the model of key interest and the most challenging model to fit in terms of the number of parameters, but the results for the other, more parsimonious, models are comparable.

**Fig. 5:**
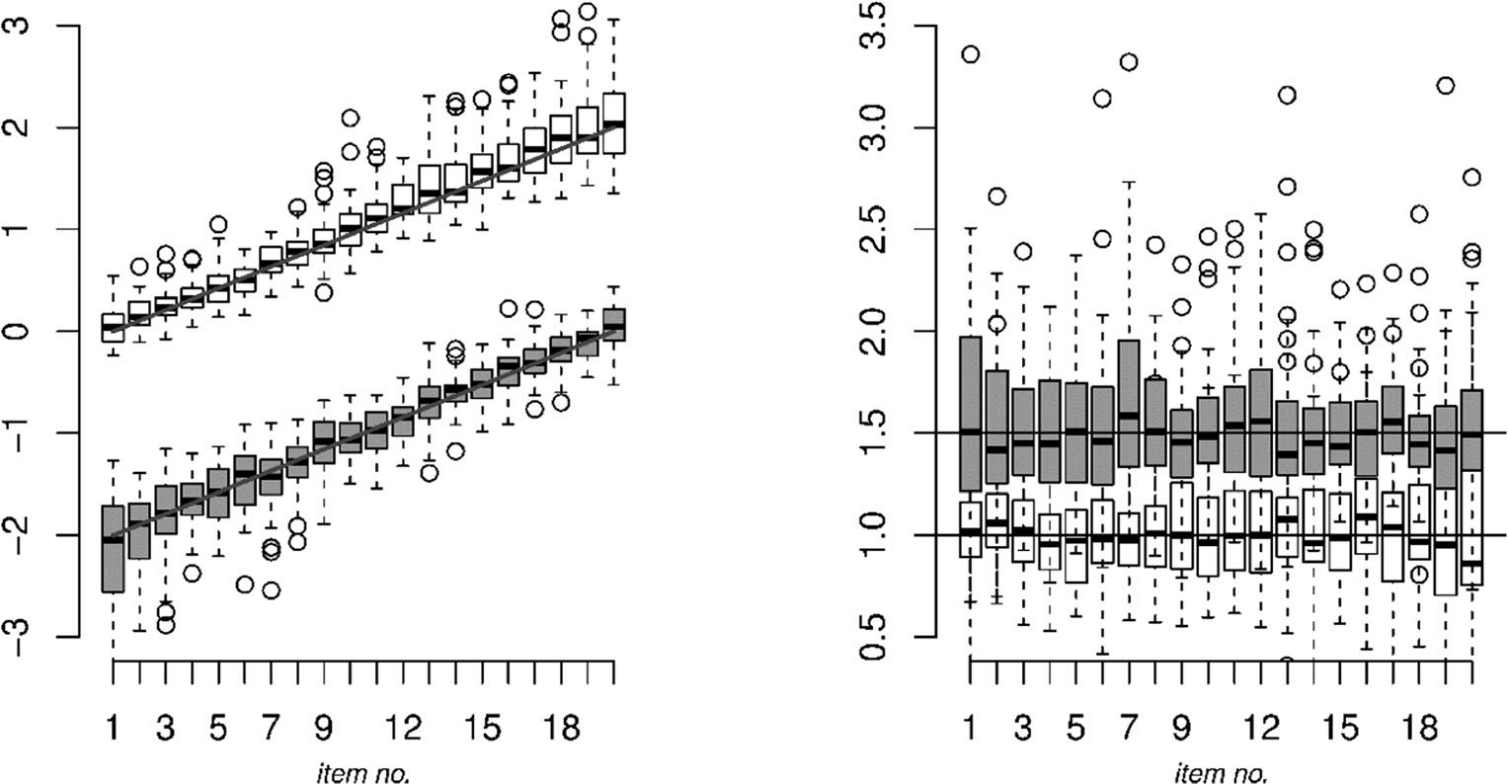
Parameter recovery for the easiness parameters (left) and discrimination parameters (right) for the two states (gray: state 0, the slower state; white: state 1, the faster state), in the presence of heteroscedasticity in the response times between states that is explicitly accounted for using the scale factor

To study the parameter recovery of the model, Fig. 5 depicts box plots of the item parameter estimates *β*_0*i*_, *β*_1*i*_, *α*_0*i*_, and *α*_1*i*_ across replications for the heteroscedastic Markov states model in the heteroscedastic Markov states scenario. As can be seen, all parameters seem to be recovered acceptably, with more variability in the discrimination parameters than in the easiness parameters. In addition, overall, the parameter estimates in state 0 (gray in the figure) are associated with somewhat more variability than the parameter estimates in state 1, as state 0 is smaller than state 1.

Statistics concerning the parameter recovery of the Markov parameters (*π*_1_, *π*_1|0_, and *π*_1|1_) and the correlation between *θ*_*p*_ and *τ*_*p*_ (*ρ*) of the heteroscedastic Markov states model in the heteroscedastic Markov states scenario is depicted in Table 2. As can be seen, all parameters seem unbiased, with acceptable sampling properties (in terms of the 95% coverage rates, and the standard deviations and RMSEs of the estimates as compared to the mean standard error), although the coverage rate of *π*_1|0_ is somewhat too small (.900 instead of .950). However, overall, we think the results do not indicate any problems with the model.

**Table 2:** Recovery results for the Markov parameters and for *ρ*

Parameter	True	MEAN(Est)	*SD*(Est)	RMSE	MEAN *SE*	Coverage
*ρ*	– . 400	– . 420	. 033	. 038	. 032	. 940
π_1_	. 667	. 661	. 085	. 085	. 073	. 940
π_1|0_	. 231	. 222	. 014	. 016	. 015	. 900
π_1|1_	. 769	. 768	. 018	. 017	. 014	. 960

“Est” denotes the estimates of the corresponding parameter across the different replications, RMSE is the root-mean squared error, “*SE*” refers to the analytical standard errors of the parameter estimates (Est), and “Coverage” refers to the 95% coverage rates

**Fig. 6:**
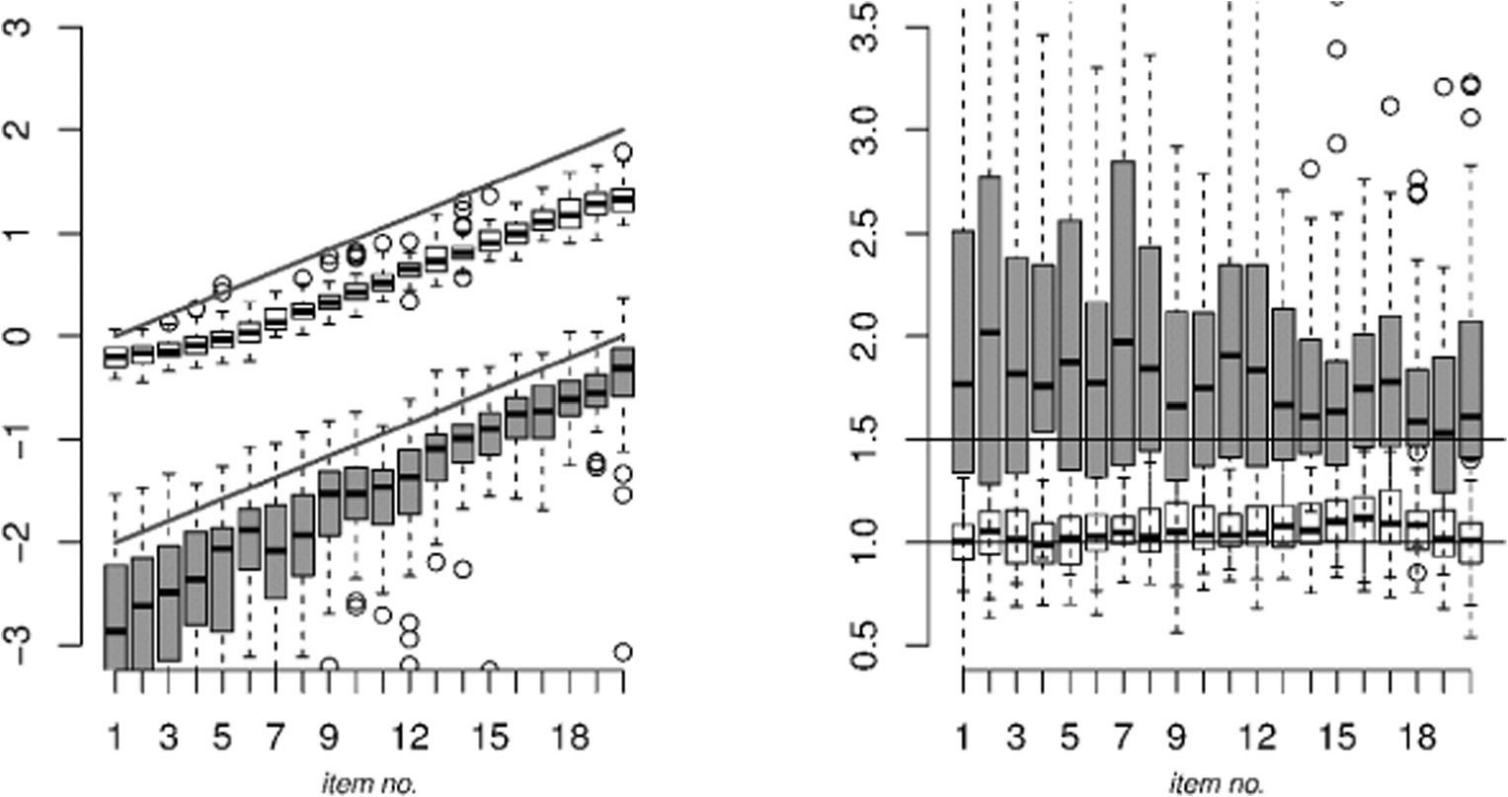
Parameter recovery for the easiness parameters (left) and discrimination parameters (right) for the two states (gray: state 0, the slower state; white: state 1, the faster state), in the presence of unmodeled heteroscedasticity in the response times

To study the effects of unmodeled heteroscedasticity between the states, Fig. 6 depicts box plots of the parameter estimates for the discrimination and easiness parameters in the heteroscedastic Markov states scenario but for the homoscedastic Markov states model. Comparing Figs. 5 and 6, it can be seen that neglecting the heteroscedasticity between states (Fig. 6) biased the parameter estimates (most notably in *α*_1*i*_, *β*_0*i*_, and *β*_1*i*_) and increased the variance of the estimates of *α*_1*i*_ and *β*_1*i*_ (as compared to Fig. 5). In addition, it can be seen that neglecting heteroscedasticity in the data decreased the variance of *α*_1*i*_ and *β*_1*i*_ as compared to the case in which heteroscedasticity was accounted for. This is due to the size of state 1 (the faster state) being overestimated: *π*_1_ has an average estimate of .816 (*SD:* .040), where the true value equaled .666. In addition, state 0 was relatively unstable: The average estimate of transition parameter *π*_0|1_ was equal to .463 (*SD:* .0452), where the true value equaled .231. State 1 was estimated to be relatively stable: The average estimate of transition parameter *π*_1|1_ was equal to .844 (*SD:* .010), where the true value equaled .769. Thus, Class 1 was still relatively stable, while Class 0 appeared relatively unstable.

#### True positive rates

See Table 3 for the detection rates of the fit indices in each data scenario. The detection rate of a given model is the proportion of replications in which that model was indicated to be the best-fitting model among the five models considered. In the table, the true positive rates of a model are marked in gray. The true positive rate of a model is the detection rate of that model in the case that the model is fit to its corresponding scenario (e.g., the baseline model to the baseline scenario).^3^ All other detection rates in Table 3 are false positives, which ideally should be close to 0. We consider true positive rates between .80 and 1.00 to indicate a good true positive rate, rates between .70 and .80 as acceptable, rates between .50 and .70 as moderate, and rates below .500 as poor.

**Table 3:**
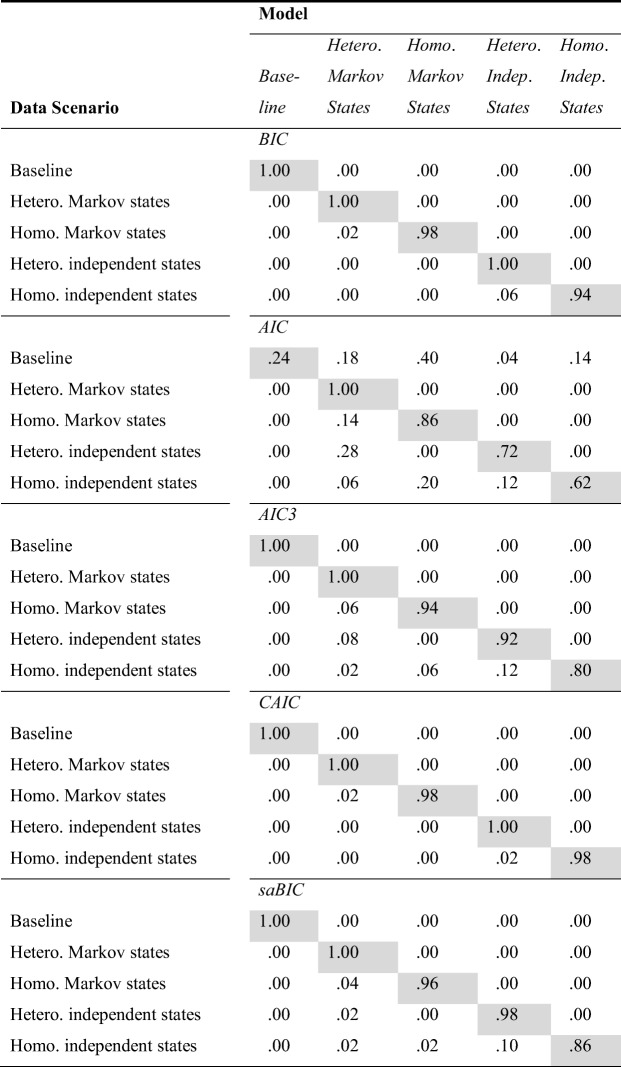
Detection rates of the BIC, AIC, AIC3, CAIC, and saBIC for the five models in each data scenario

Gray shading indicates the true positive rates (the detection rate for a model in its corresponding scenario—e.g., the baseline model in the baseline scenario); the other rates are false positive rates. In addition: “Hetero.” denotes “Heteroscedastic” and “Homo.” denotes “Homoscedastic”

As can be seen from Table 3, for the baseline model and the heteroscedastic Markov states model, true positives are perfect (i.e., 1.00) for all fit indices, but the true positive rate for the AIC is only .24 for the baseline model. As can be seen from the false positive rate in the baseline scenario, using the AIC fit index, the baseline model is hard to distinguish from the homoscedastic Markov states model, which is associated with a false positive rate of .40. For the homoscedastic Markov states model, true positives are all acceptable to good, with values between .86 and .98. For the heteroscedastic independent states model, the true positives are also considered acceptable to good, with values between .72 and 1.00, and for the homoscedastic independent states model, the true positive rate is moderate for the AIC, with a rate of .62, but acceptable to good for the other fit indices, with values between .80 and .98.

### Conclusion

In conclusion, it appeared that parameter recovery is acceptable and that all fit indices but the AIC behaved acceptably in selecting among the different models under the circumstances simulated. The poor behavior of the AIC in model selection is in line with the findings of Molenaar et al. (2016), who also found poor performance of the AIC in selecting among models that did and did not include (Markov) mixtures. In addition, we found that neglecting heteroscedasticity between classes may bias the item parameter estimates and increase their variance.

The main purpose of these simulations was a proof of principle in the sense that we wanted to show that we can adequately recover the true parameter values of the model and that we can distinguish well between the different models given a reasonable sample size and reasonable effect sizes. However, the results above depend on the choices we made concerning parameter values. That is, true positives will decrease for decreasing differences between the states in terms of *δ*_*c*_ and *β*_*ci*_ and *α*_*ci*_. In addition, if the stability of the states decreases (reflected by larger values for *π*_1|0_ and smaller values for *π*_1|1_) true positives will also decrease (see, e.g., Molenaar et al., 2016).

## Illustration

### Data

In this section, we demonstrate the viability of the present modeling approach in a real dataset. We used the responses and response times to the block design subtest of the Hungarian WAIS-IV (Nagyné Réz et al., 2008). These data have been analyzed by Molenaar, Bolsinova, Rósza, and De Boeck (2016), who analyzed these data using a mixture model for the responses but not for the response times. The data consist of the responses and response times of 978 respondents to 14 items. The items were designed to be decreasing in easiness. The raw response times are between 1 and 360 s. We omitted Item 1 from the analysis as this item caused numerical problems due to the high success rate (.999). We used the same procedure as in the simulation study. That is, we used the same categorization procedure for the raw response times, we considered the same models, and we used the same estimation procedure.

### Results

See Table 4 for the model fit indices of the models considered. As can be seen, all fit indices indicate the heteroscedastic Markov states model to be the best-fitting model. Below we discuss the results from this model. First, it appeared that Class 1 (the faster class) is somewhat larger with an initial state parameter *π*_1_ estimate of .617 (*SE*: 0.052). In addition, the classes seem relatively stable with transition parameters *π*_1|0_ and *π*_1|1_ estimated to be .124 (*SE*: 0.016) and .840 (*SE*: 0.015), respectively. In addition, *δ*_1_ was estimated to be 3.484 (*SE*: 0.210), and the residual standard deviation in Class 1, *σ*_1_, was 1.695 (*SE*: 0.131), indicating that Class 1 is associated with more variability in the response times.^4^

**Table 4:** Model fit indices for the five models considered in the application, for *T* = 5

Model	BIC	AIC	AIC3	CAIC	sBIC
Baseline	27,612	27,163	27,255	27,704	27,320
Heteroscedastic Markov states	**27,043**	**26,442**	**26,565**	**27,166**	**26,652**
Homoscedastic Markov states	27,068	26,472	26,594	27,190	26,681
Heteroscedastic independent states	27,428	26,837	26,958	27,549	27,044
Homoscedastic independent states	27,437	26,851	26,971	27,557	27,056

The best values of the fit indices are in boldface

**Fig. 7:**
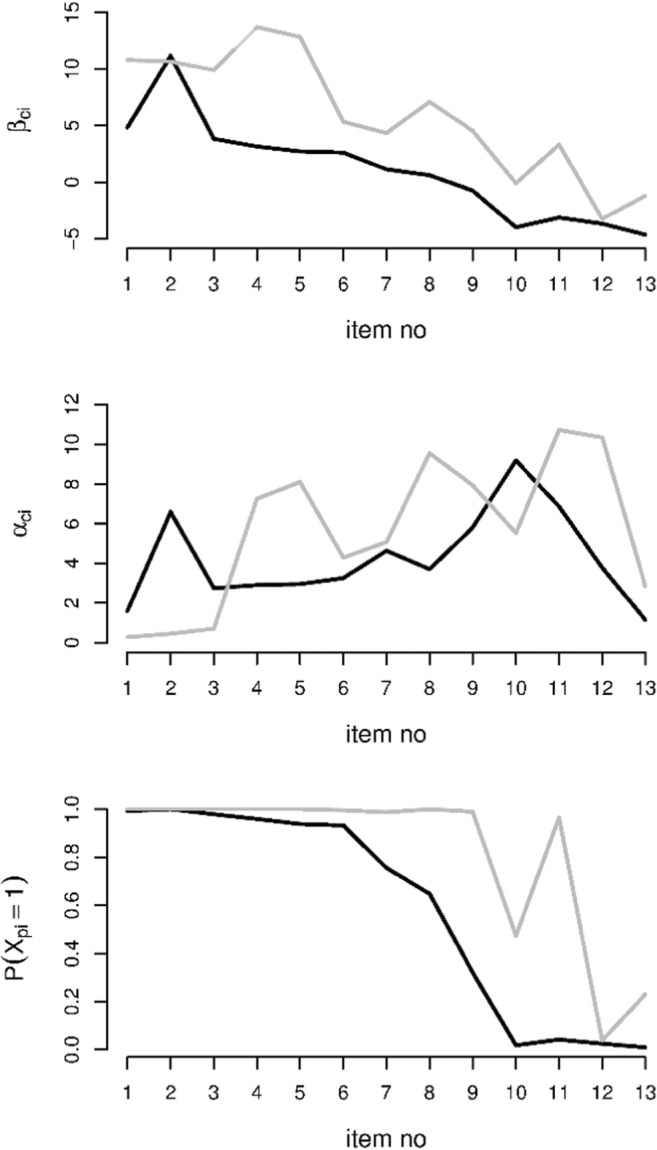
Parameter estimates for the easiness parameters (top) and discrimination parameters (middle), together with the implied marginal probabilities of a correct response in Class 0 (black lines) and Class 1 (gray lines)

In Fig. 7, the item easiness parameters, discrimination parameters, and marginal probabilities of a correct response in the two classes are plotted. As can be seen, the easiness parameters in Class 1, *β*_1*i*_, are generally larger than the easiness parameters in Class 0, *β*_0*i*_. For the discrimination parameters, there is a less clear difference: It seems that the discrimination parameters in Class 1, *α*_1*i*_, are somewhat larger than the discrimination parameters in Class 0, *α*_0*i*_, for the items later in the test (from Item 4 onward, with Item 10 as an exception), but this effect is small.

**Fig. 8:**
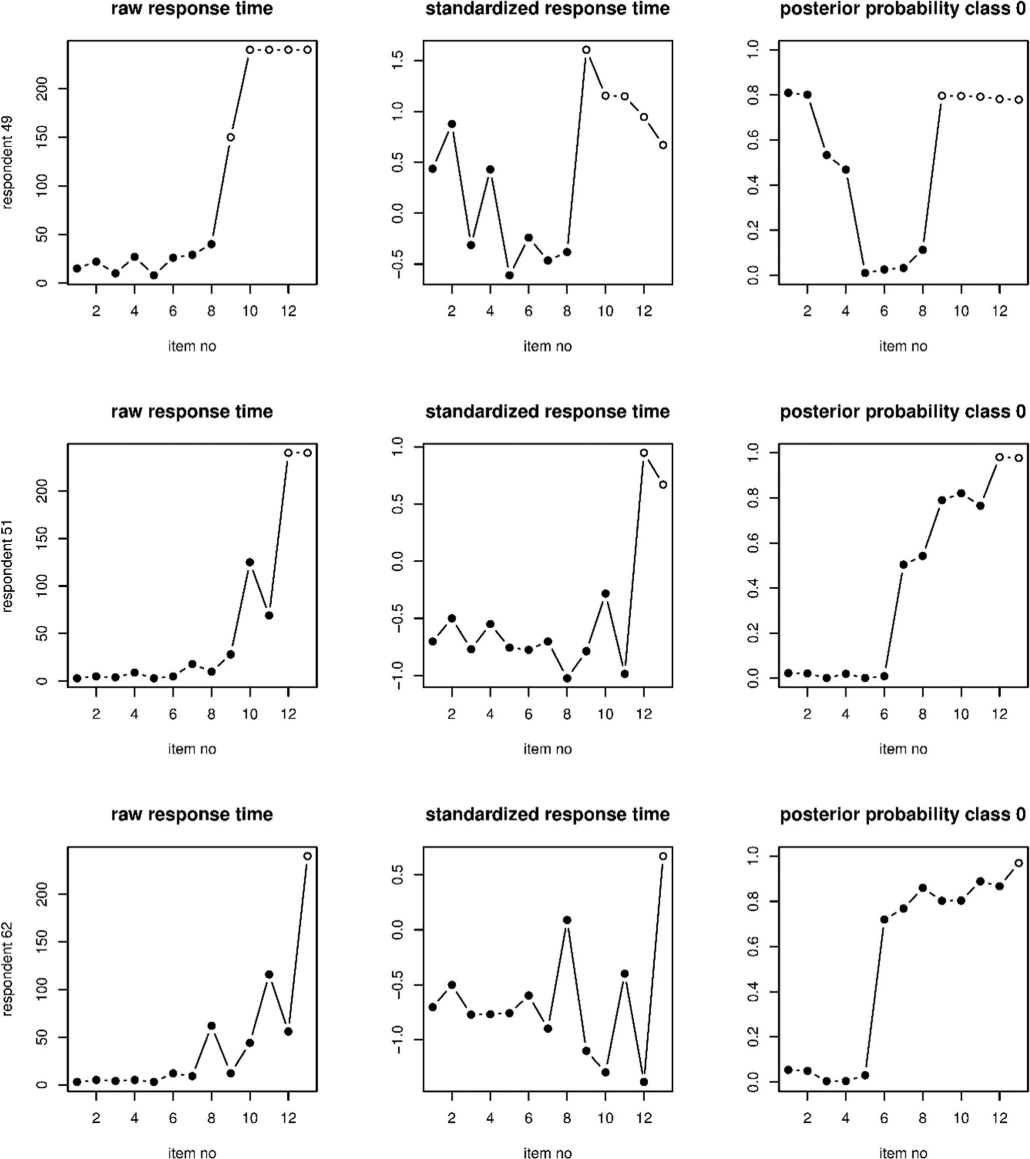
Raw response times, the item-wise standardized response times, and posterior probabilities of Class 0 for three example respondents. Solid dots denote that the response to that item was correct

Figure 8 depicts the raw response times, the item-wise standardized response times, and the posterior probabilities of Class 0 according to the heteroscedastic Markov states model for three example respondents. The raw response times are hard to interpret, as the items differ in their time intensity. The item-wise standardized response times provide an ad-hoc method to account for this confounding effect. However, besides the ad-hoc nature of this method, a drawback is that it does not account for the dependency between adjacent items and for the response outcome (correct or incorrect). As can be seen, the posterior probabilities generally give an improved picture of the response dynamics, as compared to the standardized response times, with a clearer pattern. In addition, the classification is sometimes different for the posterior probabilities than for the standardized response times. For instance, for Respondent 62, the responses to Items 9, 10, and 12 are the fastest among all items according to the standardized response times, but according to the posterior probabilities, these responses are likely in Class 0 (the slower class).

### Robustness analysis

To see whether the results above are robust to the exact number of response time categories used, we also conducted the above analyses using *T* = 3 and *T* = 2 response time categories. In the case of *T* = 3, we categorized the continuous response times of each item at percentiles 15.87 and 84.13 (obtained from a standard normal distribution at – 1 and 1). In the case of *T* = 2, we used a median split of the continuous response times of each item (i.e., we used a cutoff corresponding to percentile 50).

First, the estimates of parameters *π*_1_, *π*_1|0_, and *π*_1|1_ are .599 (*SE:* .059), .152 (*SE:* .027), and .848 (*SE:* .016) for *T* = 3, and .582 (*SE:* .067), .241 (*SE:* .018), and .759 (*SE:* .021) for *T* = 2. As we discussed above, for *T* = 5 these estimates were, respectively, .617 (*SE:* .052), .124 (*SE:* .016), and .840 (*SE:* .015), respectively. As judged by the standard errors, these estimates do not differ importantly.

**Fig. 9:**
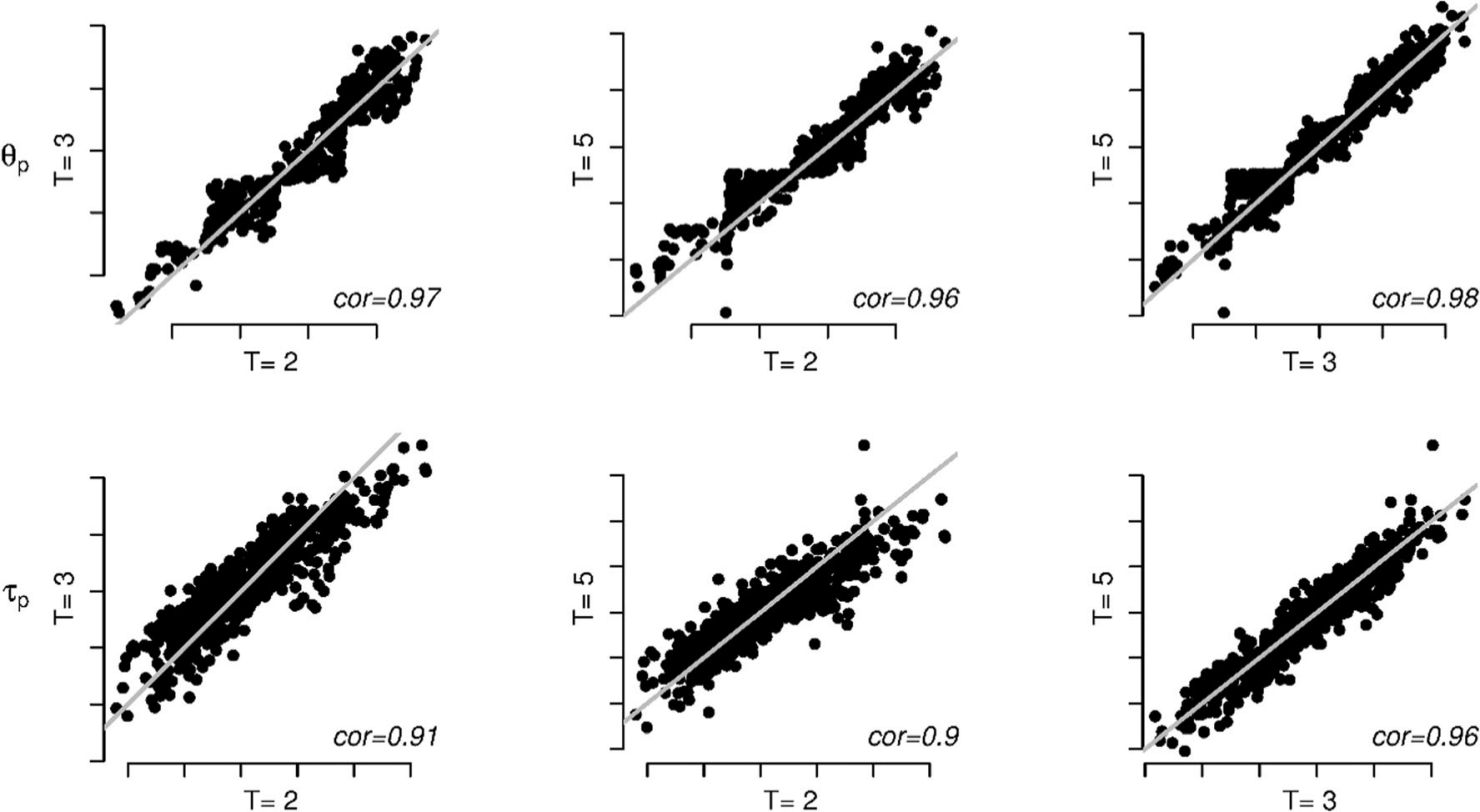
Plot of the estimates for *θ*_p_ (first row of plots) and *τ*_p_ (second row of plots) for different numbers of response time categories, *T* (left plots: *T* = 2 vs. *T* = 3; middle plots: *T* = 2 vs. *T* = 5; right plots: *T* = 3 vs. *T* = 5). The solid gray lines denote one-to-one correspondences

**Fig. 10:**
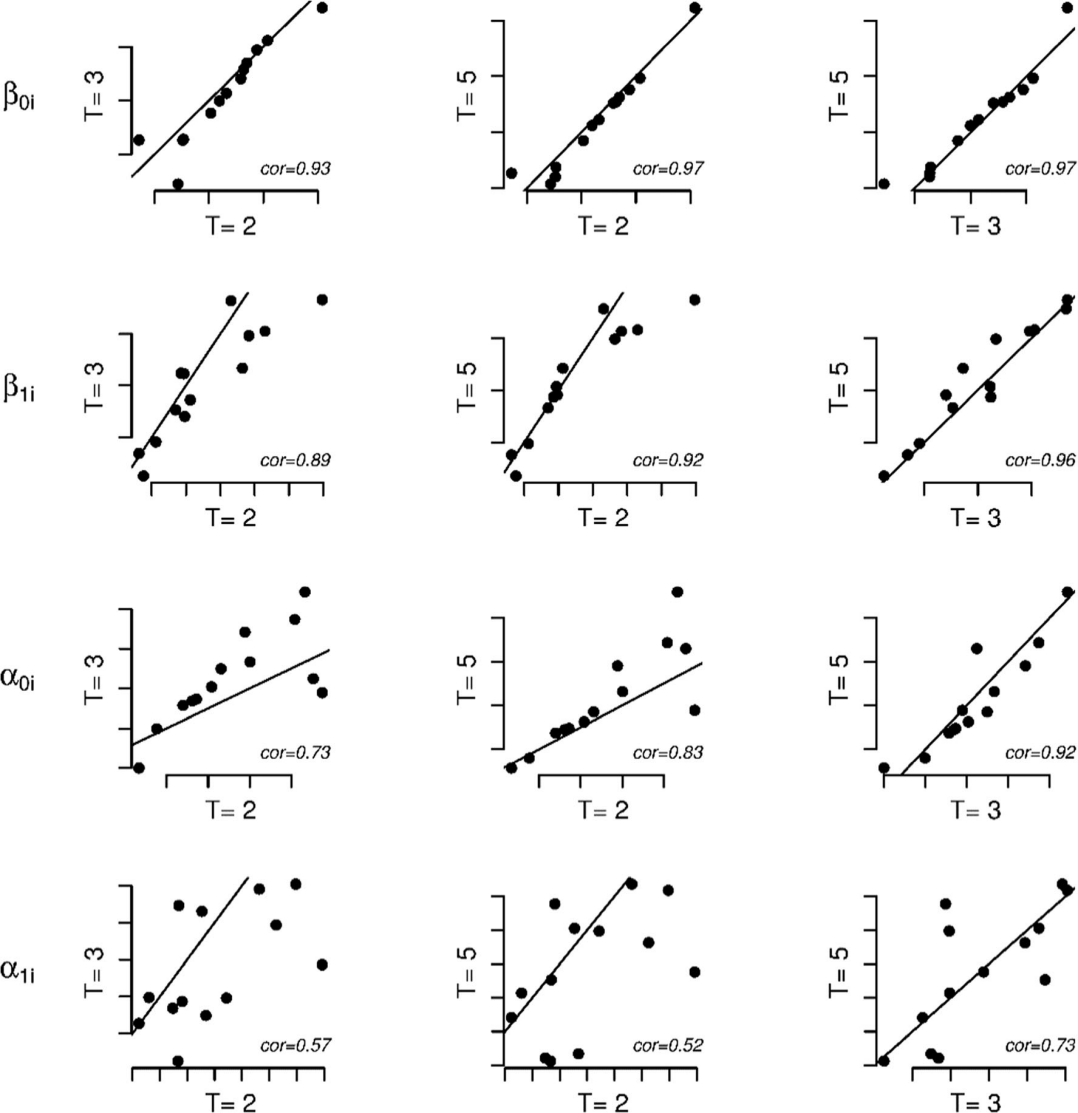
Plot of the estimates for *β*_0*i*_ (first row of plots), *β*_1*i*_ (second row of plots), *α*_0*i*_ (third row of plots), and *α*_1*i*_ (fourth row of plots) for different numbers of response time categories, *T* (left plots: *T* = 2 vs. *T* = 3; middle plots: *T* = 2 vs. *T* = 5; right plots: *T* = 3 vs. *T* = 5). The solid black lines denote one-to-one correspondences

Tables 5 and 6 contain the fit measures for the different models for, respectively, *T* = 3 and *T* = 2. As can be seen, all fit measures favor the full model in both the *T* = 3 and *T* = 2 data. This is in line with the conclusions draw above for the *T* = 5 case (see Table 4). To compare the parameter estimates from the *T* = 5, *T* = 3, and *T* = 2 data, we plotted the person parameter estimates of *θ*_*p*_ and *τ*_*p*_ (Fig. 9) and the item parameter estimates of *β*_0*i*_, *β*_1*i*_, *α*_0*i*_, and *α*_1*i*_ (Fig. 10) for the *T* = 5, *T* = 3, and *T* = 2 data. As can be seen from Fig. 9, there is a strong one-to-one correspondence between the person parameter estimates obtained from the different datasets. In Fig. 10, it can be seen that for the item parameters, the correspondence between the *T* = 5, *T* = 3, and *T* = 2 parameter estimates is best for *β*_0*i*_ and *β*_1*i*_. For *α*_0*i*_, the correspondence is associated with somewhat more noise than for *β*_0*i*_ and *β*_1*i*_. For *α*_1*i*_ the correspondence is noisiest. This has to do with the relatively large standard error of the *α*_1*i*_ parameters as compared to the other item parameters. However, for the item parameters overall, there does not seem to be a systematic difference between the parameter estimates from the different datasets. We therefore conclude that the robustness of the results across the different numbers of response time categories is acceptable.

**Table 5:** Model fit indices for the five models considered in the application for T = 3

Model	BIC	AIC	AIC3	CAIC	sBIC
Baseline	20,553	20,231	20,297	20,619	20,343
Heteroscedastic Markov states	**20,158**	**19,689**	**19,785**	**20,254**	**19,853**
Homoscedastic Markov states	20,178	19,709	19,805	20,274	19,873
Heteroscedastic independent states	20,417	19,958	20,052	20,511	20,118
Homoscedastic independent states	20,426	19,967	20,061	20,520	20,128

The best values of the fit indices are in boldface

**Table 6:** Model fit indices for the five models considered in the application for T = 2

Model	BIC	AIC	AIC3	CAIC	sBIC
Baseline	18,603	18,344	18,397	18,656	18,435
Heteroscedastic Markov states	**18,096**	**17,686**	**17,770**	**18,180**	**17,829**
Homoscedastic Markov states	18,102	17,696	17,779	18,185	17,838
Heteroscedastic independent states	18,408	18,008	18,090	18,490	18,148
Homoscedastic independent states	18,404	18,008	18,089	18,485	18,147

The best values of the fit indices are in boldface

## Discussion

In this article, we presented a mixture model to detect heterogeneity in the response processes underlying psychometric test items. The new model combines the strengths of previous mixture models by Schnipke and Scrams (1997), Wang and Xu (2015), Wang et al. (2018), Molenaar et al. (2016), and Molenaar et al. (2018). In our modeling approach we used mixture modeling in an indirect application (Yung, 1997). That is, the mixture components in our model are not necessarily substantively interpretable but are rather statistical tools to detect heterogeneity in the data that is due to differences in response processes. This is different from the modeling perspective by for instance Wang and Xu who used mixture modeling in a direct application (Dolan & van der Maas, 1998) in which the mixture components are substantively interpreted. Specifically, Wang and Xu distinguished between a fast guessing process and a solution process. Regardless of the nature of the mixture application (direct or indirect), the methodology presented in this article is equally amenable to the modeling of fast guessing and solution behavior. That is, if the measurement model for the responses in the faster state is restricted to represent fast guessing (i.e., discrimination equal to 0, see Table 1), the model is in essence the model by Wang and Xu, but with Markov-dependent states. Other restrictions are possible, which we will illustrate below. However, such restrictions need a strong theory about the response processes, which is not always available.

Throughout this article, we have assumed two latent states to underlie the item responses and response times, this has mainly a pragmatic reason in the sense that we think that two states can capture the most important patterns in the data. In addition, some theories describe binary processing, for instance the automated versus controlled processing theory (Shiffrin & Schneider, 1977), and the fast versus slow intelligence theory (DiTrapani, Jeon, De Boeck, & Partchev, 2016; Partchev & De Boeck, 2012). However, it can certainly be that some situations require more than two states (e.g., if three clearly distinct solution strategies underlie the response behavior of the respondents). In principle, it is straightforward to extend the present model to include three or more item specific states. However, the number of parameters rapidly grows. That is, for three item specific states, six parameters need to be estimated for each response variable (three discriminations and three easiness parameters). In such a situation, either the sample sizes should be very large, or one should incorporate reasonable model restrictions. That is, model restrictions can be thought of that are either pragmatically defendable or that are derived from theory. For instance, Molenaar et al. (2018) considered a model in which the item parameters have an overall difference across states and not an item specific difference (as in the models considered in the present article). In addition, Molenaar et al. (2016) used the restrictions that van der Maas and Jansen (2003) derived from the developmental theory by Siegler (1981) to distinguish different solution strategies underlying the Piagetian balance scale task. Using these restrictions, Molenaar et al. (2016) identified up to five states in a hidden Markov model for responses and continuous response times.

To solve the problem of spurious mixtures, we followed Molenaar et al. (2018) and categorized the continuous response times. This approach is pragmatic but shown effective in countering false positives in the case of distributional misfit. However, the approach has the drawback that information about individual differences is decreased such that the power to detect an effect may depend on arbitrary choices concerning the number and location of the cut-off values. It is therefore advisable to always investigate the robustness of the results with respect to the cut-off values as was illustrated in our real data example.

Another aspect of the general mixture modeling framework considered in this article (Table 1) is the operationalization of response processes in terms of the item properties (discrimination and easiness) and the expected response times. That is, a response process difference is assumed to be characterized by (1) a difference in the discrimination and/or easiness parameter and (2) a difference in the expected response time. This operationalization in Difference 1 can be justified by the statistical theory about measurement invariance (Mellenbergh, 1989; Meredith, 1993), which dictates that a difference in measurement model parameters indicates a difference in the interpretation of the underlying latent variable. That is, if faster responses are associated with different measurement parameters (discrimination and/or easiness) as compared to the slower responses, the latent variable has a different interpretation for these responses indicating a different response process. As we discussed before, the operationalization in Difference 2 can be justified by the theory about response times in experimental psychology (e.g., Luce, 1986), which dictates that the response times indicate the time that is needed for a certain psychological process to be executed. A difference in expected response time thus indicates a different process (all other things being equal).

An alternative to the statistical operationalizations of response processes adopted here are process-modeling operationalizations from mathematical psychology. In this framework, stronger assumptions are made about the response process (e.g., a response process consists of noisy information accumulation that stops if enough information for one of the response alternatives is gathered). From these assumptions, a mathematical model can be derived that is fit to the data. Examples of such models include the diffusion model (Ratcliff, 1978), the linear accumulator model (Brown & Heathcote, 2008), and the race model (Audley & Pike, 1965). However, these models are mathematically more complex, which made them less suitable to the aims of the present article. Yet it will certainly be interesting to consider models from mathematical psychology in light of the present mixture modeling framework.

## Appendix

The syntax below can be used to fit the heteroscedastic hidden Markov mixture model to responses and categorized response times in LatentGOLD.

## Footnotes

1 Note that the restrictions provided in Table 1 result in models equivalent to the models discussed in the text [i.e., equivalent in terms of the likelihood of the model. The exact parameterization in the corresponding articles is for some cases slightly different. For instance, Schnipke and Scrams (1997) estimated ln(ν_ci_) instead of *ν*_*ci*_, and Wang and Xu (2015) used *α*_1*i*_(*θ*_*p*_ – *β*_1*i*_) in the three-parameter model, instead of *α*_1*i*_*θ*_*p*_ + *β*_1*i*_.

2 However, note that it is possible to specify the three-parameter model as a mixture of a two-parameter model and a guessing model, which is a generalized linear model.

3 Note that we cannot speak of the “true model,” because the response time data were simulated under a different model (a categorized Box–Cox-transformed log-normal model) from the model applied to the data (a partial-credit model; see Eq. 11).

4 For numerical reasons, we estimated the logits of the initial-state and transition parameters. In addition, we estimated *exp*(*– σ*_1_). The reported standard errors were obtained using the delta method.
